# Supplementation with dairy calcium and/or flaxseed fibers in conjunction with orlistat augments fecal fat excretion without altering ratings of gastrointestinal comfort

**DOI:** 10.1186/s12986-017-0164-8

**Published:** 2017-02-07

**Authors:** Mette Kristensen, Signe Rømer Juul, Karina Vejrum Sørensen, Janne Kunchel Lorenzen, Arne Astrup

**Affiliations:** Department of Nutrition, Exercise and Sports, Faculty of Science, University of Copenhagen, Rolighedsvej 26, Frederiksberg, DK-1958 Denmark

**Keywords:** Orlistat, Flaxseed, Calcium, Side effects, Weight loss, Obesity

## Abstract

**Background:**

Orlistat is a lipase inhibitor which reduced absorption of dietary fat by ~30% thereby inducing a weight loss; however, side effects occur as a consequence of increased colonic fat content. To test the hypothesis that most gastrointestinal side events induced by treatment with orlistat could be prevented/ameliorated by concomitant use of natural dietary components, flaxseed fiber (FF) and/or dairy calcium (Ca), binding liquid fats to more solid complexes.

**Methods:**

A randomized controlled dietary intervention study. Thirty-eight obese adults completed a 1-week run-in period, where all participants were treated with orlistat (60 mg t.i.d) and were hereafter randomized to 12 weeks dietary supplementation with/without 5 g FF (FF+/FF-) and/or 1200 mg dairy calcium (Ca+/Ca-) in conjunction with orlistat. All feces were collected for 3 days, and diet was recorded for 5 days, during run-in and week 4. The primary end-point, gastrointestinal symptoms, was assessed biweekly. At baseline and after 12 weeks, cardiometabolic risk markers and anthropometrics were evaluated as secondary end-points.

**Results:**

Both FF and Ca increased fecal fat excretion (*P* = 0.02 and *P* = 0.04, respectively). Although fecal fat excretion increased by ~100% in the FF+/Ca + group, and only by ~12% in the FF-/Ca + group, no interaction between FF and Ca was present, suggesting an additive effect. The fecal fat excretion was ~10 g/d higher with FF and Ca (~25 g/d) compared to fecal fat excretion with orlistat alone (~15 g/d). Mean ratings of severity of diarrhea tended to increase with Ca (*P* = 0.03) but not with FF. No other gastrointestinal symptoms, or a composite score of symptoms, were affected by the dietary supplements. Body weight was reduced in all groups but did not differ between groups, whereas waist circumference was most reduced in the FF+/Ca + group. No effects of dietary supplements on cardiometabolic risk factors were observed, except a slight increase in diastolic blood pressure (*P* = 0.03) with FF, but not Ca.

**Conclusions:**

Our results do not support an improvement in orlistat-induced gastrointestinal side effects by concomitant use of FF and Ca. However, fecal fat excretion was increased with both FF and Ca in the absence of a worsening of symptoms, warranting further studies powered to detect potential additive weight loss effects.

**Trial registration:**

Ethical Committee of the Capital Region of Denmark reg. no. H-1-2010-110, 02-11-2010 database no. NCT01320228, 21-03-2011.

**Electronic supplementary material:**

The online version of this article (doi:10.1186/s12986-017-0164-8) contains supplementary material, which is available to authorized users.

## Background

Orlistat is a gastrointestinal lipase inhibitor and thus reduces the extent of fat absorption from the diet. At the recommended therapeutic dose (60–120 mg t.i.d.) and in conjunction with dietary advice, orlistat produces a negative energy balance which results in weight loss of 2.9 kg (95% CI: 2.5; 3.2) more than placebo [1]. Orlistat inhibits absorption of dietary fat by ~30%; however, side effects occur as a consequence of increased colonic fat content. The most commonly reported side effects are oily or fatty stools, oily discharge, fecal incontinence as well as abdominal pain, nausea, vomiting, diarrhea and rectal pain [2]. They are usually temporary; however, most patients do not use orlistat long enough to experience a reduction in gastrointestinal symptoms as also substantiated by the increased risk of discontinuation when treated with orlistat compared to placebo reported in a meta-analysis, where ~40% of dropouts were related to gastrointestinal symptoms [3]. Thus, in order to optimize the treatment of orlistat, ways to curb the gastrointestinal side effects for orlistat are of great interest.

With orlistat 120 mg t.i.d., the proportion of ingested fat being excreted in stool have been found to be increased from baseline levels of 6% to between 20 and 35%, with an estimated mean value of 30% [4]. With a lower dosage of orlistat around 20% of the ingested fat is daily excreted depending on body weight, degree of caloric restriction and the proportion of fat in the diet [4]. We have previously found that increasing dietary calcium intake from e.g. 500 to 2000 mg is sufficient to bind 5–7 g of fat [5–7], and although a meta-analysis was unable to find difference in magnitude of effect whether calcium originated from dairy or other sources [8], we recently found dairy calcium to result in a greater fecal fat excretion compared to supplemental calcium, and to better diminish postprandial chylomicron triacylglycerol concentration [9], which is also indicative of an interference with fat absorption. Although the mechanism of action is not fully elucidated, it has been proposed that calcium and lipids form insoluble soaps in the intestine, and thus, an increased fecal fat content resulting from increased calcium intake may not give rise to gastrointestinal symptoms. Likewise, we have found flaxseed fibers (FF) to inhibit fat absorption, even when administered in small doses in humans [10–12], as well as in an animal model [13], likely through a binding and/or encapsulation of fat in the intestine [14]. Dietary fibers, particularly those which form viscous solutions when hydrated, may encapsulate fat in a more viscous chyme, but in vitro studies have also suggested that dietary fibers to bind directly to lipids and bile acids [15]. Thus, dietary fibers may also affect fecal fat excretion. However, one previous study, in which interaction between intake of dietary fat and fiber and orlistat was investigated, fecal fat excretion was not altered by high vs. low fiber diets [16]. However, accessibility of dietary fat (intracellular vs. extracellular fat) did influence the fat-blocking actions of orlistat. Further, Cavaliere et al. [17] previously found that daily supplementation with 12–18 g of psyllium over 30 days reduced gastrointestinal symptoms compared to placebo; however fecal fat excretion was not investigated.

In addition to its effect on fat-absorption, flaxseed fibers also suppress hunger [11, 18], and thus may aid weight loss when given in conjunction with orlistat, whereas the evidence for an effect of calcium on appetite is more controversial [19].

Here, we test the hypothesis that supplementation with flaxseed fibers and/or dairy calcium in the form of Capolac® (Ca) will alleviate the gastrointestinal symptoms related to orlistat treatment. Furthermore, the effects of dietary supplementation in conjunction with orlistat on fecal parameters, dietary intake, anthropometry, cardiometabolic risk markers and adverse events were evaluated.

## Methods

### Study design

The study was designed as a randomized controlled parallel intervention study of 13 weeks duration. For one week (run-in), all participants received alli® (Orlistat, 60 mg t.i.d) and dietary counseling to facilitate a weight loss. Hereafter participants were randomized in a 2 × 2 factorial design to supplementation with/without flaxseed fibers and dairy calcium in addition to treatment with alli® for another 12 weeks. Anthropometry, blood pressure and gastrointestinal symptoms was assessed every 2 weeks from run-in to week 12, fecal samples were collected for 72 h during run-in (alli® only) and during week 4 (alli® plus dietary supplements) concurrent with a 5d weighed food record. Fasting blood samples were drawn at time of randomization (week 0) and at the end of the study (week 12). At each visit, other adverse events were registered as part of an interview.

### Randomization and allocation concealment

Randomization was done using a stratified block randomization according to sex (M/F) and body mass index (BMI) (BMI < 35 kg/m^2^ ≥ BMI). Within each of the four strata, participants were randomized to supplementation with/without Ca and FF, respectively, by block randomization using web-based software (www.randomization.com) with uneven sized blocks. The randomization list was created by one of the research team prior to commencement of the study (MK), and allocation of supplement group was done by one of the research team (MK, SR, KVS) during the randomization visit (week 0), thus allocation was not concealed.

### Participants

Inclusion criteria were BMI between 30 and 40 kg/m^2^ and age between 20 and 60 years. Exclusion criteria were: dairy allergy and/or intolerance, allergy to Orlistat, infectious or metabolic diseases, gastrointestinal disorders (previous and current), dietary supplement use during the study and 1 month prior to the study, >1 y postmenopausal (self-reported), pregnancy or lactation, previous use of Orlistat, treatment with medication known to interact with orlistat, dieting or weight change >3 kg within 3 months prior to the study, and participation in other studies. Prescription medication was considered on an individual basis. All participants attended a screening visit before enrolment, where weight, height, waist circumference and blood pressure were measured. Also, they were interviewed to assess eligibility for enrolment. Here, they also filled in a Three Factor Eating Questionnaire, in which each item scores either 0 or 1 point. The minimum score for factors I-II-III is therefore 0-0-0, the possible maximum score 21-16-14 [20].

### Experimental diet

#### Weight loss program

The participants followed a slightly energy-restricted diet based on their habitual choice of food items to aid weight loss. The weight loss program was based on an educational system, consisting of five color-coded iso-energetic interchangeable units of 250 kJ representing different nutrients (protein-rich, complex carbohydrate-rich, simple carbohydrate-rich, fat-rich and alcohol). The participants were instructed to adhere to a diet of at least −1250 kJ/d less than their estimated energy requirement (ER) [21], but no less than 5000 kJ/d. To ensure an even distribution of fat in the meals, the participants were instructed to consume a minimum 20 g of fats with every meal. Also, they were instructed to a minimum intake of protein of 60 g/d to minimize loss of lean body mass. ER was assessed based as follows: ER (kJ/d) = body weight × X + 4500), where X = 47 for women and 63 for men. The participants met with a dietitian 4 times during the study, and were instructed to keep a food diary which included count of total units and number of color-coded units with special emphasis on fat and protein in order to adhere to the instructions provided.

#### Dietary supplements

The participant were instructed to take alli® (60 mg orlistat t.i.d) in conjunction with their main meals for 13 weeks in total. After a 1-week run-in period on alli®, the participants were randomized to supplementation with/without flaxseed fibers and dairy calcium in addition to treatment with alli®. The supplements were given three times daily together with the main meals. The supplements were provided in small sachets as powders every two weeks, and the participants were instructed to consume it either as dissolved into a beverage or sprinkled on top of their foods. The flaxseed fiber extract (Biogin biochemicals co. Ltd, China) contained 70% of dietary fiber (primarily soluble dietary fibers) and contributed 5 g of dietary fiber/d. The dairy calcium extract (Capolac® MM-0525; Arla Foods, Denmark) contained 25% Ca from milk and a total of 1200 mg of Ca was provided daily. Whole-grain rice flour served as a placebo and contributed 0.2 g of dietary fiber/d. The four different supplements were marked as A, B, C and D. This was done by the packaging company, and the supplement codes were kept in a sealed envelope by a staff member not related to the study until the study was finalized. Thus, both participants and all study staff were blinded to the coding of the supplements.

As orlistat affects fat absorption, absorption of fat-soluble vitamins may be diminished. Thus, all participants were provided with vitamin A (900 μg, 3000 iu), D (10 μg); E (335 mg) and K (150 μg) for daily supplementation (Natur-Drogeriet, Hoerning, Denmark).

### Compliance assessment

Compliance to both alli® and dietary supplements (not fat-soluble vitamins) were assessed by counting returned unused medication and dietary supplements at each visit. Participants were considered compliant when at least 85% of the medication and dietary supplement, respectively, were taken over the entire study period.

### Anthropometric measures and blood pressure

All measurements were performed in the morning after an overnight fast (>10 h) and abstention from alcohol and physical exercise for 24 h. Body weight was measured on an electronic scale while the participants were wearing light clothing and no shoes (Tanita BWB-600, Japan). Height was measured to the nearest 0.5 cm by using a wall-mounted stadiometer without shoes at the first visit (Seca, Hultafors, Sweden). Waist circumference was measured two times to the nearest 0.5 cm at the narrowest point between the iliac crest and the lowest rib after expiration using a non-flexible measuring tape and the mean value calculated. Blood pressure measurements on the same (dominant) arm at each visit were performed in the upright sitting position after 10 min of rest with an automatically inflated cuff (UA-787; A & D Co Ltd, Saitama, Japan). Two measurements with at least one minute in between were performed and the mean value calculated.

### Assessment of gastrointestinal symptoms

To assess gastrointestinal symptoms, visual analogue scales were used. They are 100 mm in length with words anchored at each end. These were used to asses both severity (0 mm = ‘none’ and 100 mm = ‘very severe’) and frequency (0 mm = ‘not more often than normal’ and 100 mm = ‘much more often than normal’) of 15 different gastrointestinal symptoms. These symptoms were selected based on known adverse events related to orlistat treatment, and includes: oily stools, flatus with oily discharge, fecal incontinence, fecal urgency, loose stools, frequent stools, bloating, nausea/vomiting, rectal pain, abdominal pain, stomach rumbling, flatus, oily spotting, diarrhea and constipation. Gastrointestinal symptoms were assessed at each visit. Furthermore, a composite score of gastrointestinal symptoms most frequently (seen in ≥1 in 10 according to the product leaflet) observed for both severity and frequency was calculated as the mean follows: ΣFrequency _(oily stools; flatus; fecal urgency, fecal incontinence, flatus with oily discharge, loose stools, abdominal pain, diarrhea, frequent stools)_/9 and ΣSeverity _(oily stools; flatus; fecal urgency, fecal incontinence, oily spotting, loose stools, abdominal pain, diarrhea, frequent stools)_/9.

### Assessment of dietary intake

A food frequency questionnaire (FFQ) to estimate the participants’ habitual Ca intake was filled in at baseline [22]. Here, habitual fiber intake of the subjects was also estimated using a FFQ, in which participants stated portion size and frequency for their intake of fiber-rich food items during the last month. The habitual daily dietary fiber intake was estimated by using the average fiber content of a range of products within each food item category. The FFQ was validated in 125 individuals against a 7-day weighed food record (Pearsons correlation 0.63; *P* < 0.001) [23].

During the run-in period, the subjects completed a 5-day weighed food record. This was done concurrent with the fecal sample collection, as the 72 h of fecal samples were collected during the last 3 days of food recording allowing for estimation of relative excretion of fat and energy. All recorded foods and energy-containing beverages were entered into the Dankost 3000 dietary assessment software (Dankost 3000, version 2.5, Danish Catering Center, Herlev, Denmark) and mean total intake of energy, fat, carbohydrates, protein and total dietary fiber was calculated for each 5-day registration period.

### Laboratory procedures

#### Blood samples

Fasting blood samples were collected at randomization (week 0) at the end of the study (week 12) after an overnight fast (>10 h) and abstention from alcohol and physical exercise for 24 h. Samples were stored at −20 °C until analyses. Plasma concentrations of glucose and serum concentrations of triacylglycerol, total, HDL and LDL cholesterol and insulin were measured as described elsewhere [11]. HOMA index of insulin resistance (HOMA-IR) was calculated as (fasting glucose (mmol/L) × fasting insulin (mU/L))/22.5. Hemoglobin A1C (HbA1c) was measured by using an immunoturbidimetric assay with a Unimate HbA1C test kit (Roche Diagnostics) on a COBAS MIRA Plus chemistry analyzer (Roche Diagnostic Systems Inc). Serum high sensitivity CRP (hsCRP) was measured by a solid-phase chemiluminescent immunometric assay using an IMMULITE® 1000 Automated Immunuassay Analyzer. All samples were analyzed in one batch after the study was completed and all CV% were <5%.

#### Fecal samples

All fecal samples were collected in pre-weighed plastic containers for 72 h during run-in (alli® only) and during week 4 of the intervention period (alli® plus dietary supplements). Participants kept the plastic containers in a cooled box and brought them to the department daily. Samples were weighed and freeze-dried, homogenized and samples from each participant’s 72 h collection were pooled. Fecal energy excretion was determined using bomb calorimetry (IKA- calorimeter system 4000, Heitersheim, Germany). Samples were hydrolyzed with 3 N hydrochloric acid before total fat content was measured by acidic Bligh & Dyer extraction [24]. The apparent digestibility of fat was calculated relative to intake as 100 × mean fat excreted (g/d)/mean intake of fat (g/d).

### Statistical analyzes

The study was a pilot study, exploratory in nature and thus no power calculation has been made. A total of 72 participants was intended to be randomized to the four treatment groups in a balanced design (*n* = 18). As side effects are expected to occur in 20–25% of the participants on treatment with alli®, it is expected that a decrease in side effect frequency and severity was detectable when summarizing the reported side effects.

All statistical analyses and calculations were performed using the Statistical Analysis System software package, version 9.3 (SAS Institute Inc., Cary, NC, USA). All dependent variables were controlled for homogeneity of variance and normal distribution by investigation of residual plots and normal probability plots. A one-way ANOVA was used to compare baseline values between groups.

An ANCOVA was used to investigate the effect of dietary supplementation on all parameters. Participant was included as a random variable, age and corresponding baseline value were modeled as covariates and FF, Ca, their interaction (FF × Ca) and sex were modeled as fixed variables. The FF × Ca interaction was only omitted, when *P* > 0.10. Posthoc pairwise comparisons between dietary supplements were made when the FF × Ca interaction was significant; otherwise main effects of FF and Ca are reported. All analyses were completed as completers only (CO), as efficacy was the primary focus of the present study. Results are presented as means ± SEM or as median (95% CI) when appropriate and the statistical significance level was defined as *P* < 0.05.

## Results

**Fig. 1:**
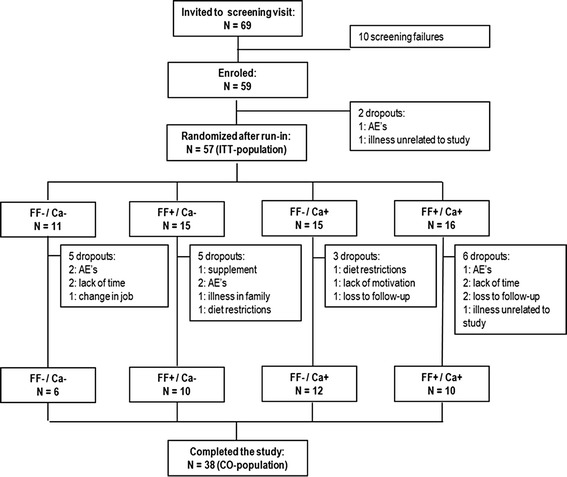
Flow diagram: Parallel intervention study with orlistat treatment plus placebo supplement (FF-/Ca-) or flaxseed fibers (FF), and/or dairy Ca (Ca) supplements as part of an energy-restricted diet. The diagram includes detailed information about the study

A total of 69 overweight and obese adults were screened for eligibility following advertising in the local papers in the Copenhagen area and pre-screening via telephone from April 2011 to January 2012. Of these, 59 were enrolled in the study, 57 were randomized after the run-in period, and a total of 38 completed the study (Fig. 1). We aimed at enrolling 72 participants; however, difficulty with recruitment resulted in a smaller sample size than initially anticipated. In total, 6 participants dropped out due to the side effects of alli® treatment, of which 1 dropped out during the run-in period, i.e. before randomization. The remaining dropouts appeared to be unrelated to the dietary intervention. There was no difference between groups in baseline characteristics (Table 1).

**Table 1:** Characteristics of the participants at randomization

	FF-		FF+	
	Ca- (*n* = 11)	Ca + (*n* = 15)	Ca- (*n* = 15)	Ca + (*n* = 16)
Age (y)				
Females/males (n)	9/2	13/2	12/3	12/4
Smokers (%)	36	40	33	38
Habitual Ca intake (mg/d)	1196 ± 533	1106 ± 517	832 ± 413	1084 ± 742
Habitual DF intake (g/d)	23.6 ± 10.3	26.2 ± 11.2	21.5 ± 6.4	22.2 ± 13.1
TFEQ:				
Cognitive dietary restraint score	7.5 ± 3.4	9.2 ± 4.2	9.4 ± 3.4	8.9 ± 3.8
Dis-inhibition score	11.0 ± 3.1	9.4 ± 3.3	9.8 ± 4.3	8.1 ± 2.7
Susceptibility to hunger score	8.7 ± 3.2	7.9 ± 3.7	8.0 ± 3.9	6.5 ± 3.8

Abbreviations: *DF* dietary fiber, *TFEQ* three factor eating questionnaire

During the study period, four participants suffered from illness which required antibiotic treatment (1 in FF-/Ca-; 2 in FF+/Ca-; 1 in FF-/Ca+). Adverse events not directly related to the gastrointestinal tract, such as headache was reported in 2, 3, 2 and 4 participants in the FF-/Ca-, FF+/Ca-, FF-/Ca + and FF+/Ca + groups, respectively and dizziness was reported in one participant in each of the FF-/Ca + and FF+/Ca- groups. Other adverse events reported included only normal illness, such as common cold, back pain, sinus infection and tooth ache, which was not a plausible side effect of the dietary supplements or alli®.

### Compliance and dietary intake

Generally, the compliance to the dietary supplements over the entire study was good (FF-/Ca-: 81.8 ± 12.8%; FF+/Ca-: 84.5 ± 12.9%; FF-/Ca+: 89.6 ± 11.2%; FF+/Ca+: 89.2 ± 4.5%). There was a tendency towards an increased compliance for Ca + vs. Ca- (*P* = 0.09), whereas compliance did not differ for FF+ vs. FF- (*P* = 0.66). In total, 8 of the 38 participants completing the study had a mean compliance below 85%. Compliance with alli® treatment was better than for dietary supplement compliance (FF-/Ca-: 95.0 ± 15.7%; FF+/Ca-: 95.9 ± 7.3%; FF-/Ca+: 99.0 ± 5.8%; FF+/Ca+: 97.7 ± 8.7%), but did not differ between groups. Only one participant had a mean compliance below 85% of alli®. Participants reported that non-compliance most often occurred in the morning. Compliance with the dietary supplements and alli® exhibited a weak, although significant correlation (Pearson’s correlation coefficient *R* = 0.39; *P* = 0.016).

There was no difference between groups at baseline in total energy intake, absolute fat intake or dietary macronutrient composition (Additional file 1: Table S1). As intended, the energy contribution from fat was high, ranging from 32.9 ± 1.6E% to 39.2 ± 3.2E% at baseline. There was no change in dietary intake over the course of the study in either group.

### Gastrointestinal symptoms

**Fig. 2:**
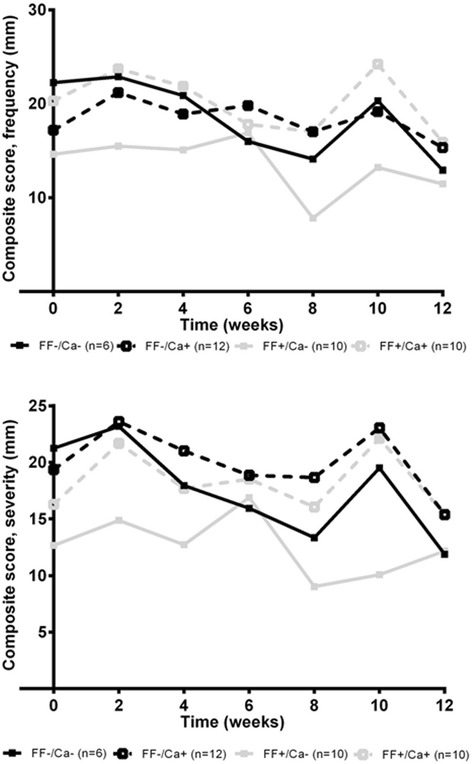
Composite score for gastrointestinal symptoms. Mean frequency and severity of composite score of 9 gastrointestinal symptoms (oily stools; flatus; fecal urgency, fecal incontinence, flatus with oily discharge, loose stools, abdominal pain, diarrhea, frequent stools) during the 12 weeks intervention with orlistat plus placebo (FF-/Ca-) or flaxseed fibers (FF) and/or dairy Ca (Ca) supplements. The lower the score the less frequent or severe the symptom

Baseline ratings of frequency and severity of gastrointestinal symptoms did not differ between groups, except for severity ad frequency of abdominal pain (Additional file 1: Tables S2 and S3). No effect of FF or Ca on neither severity or frequency of the composite score was observed (Fig. 2). Also, none of the individual gastrointestinal symptoms measured were affected by FF and Ca supplementation, except for severity of diarrhea, for which an interaction between FF and Ca tended to be present (*P* = 0.082). Posthoc analyses showed a ~6 fold increase in severity of diarrhea after combined supplementation of FF and Ca (FF+/Ca+) compared with baseline, which differed from the group only supplemented with FF (FF+/Ca-) (*P* = 0.03), thus an effect of Ca only seen with concomitant FF supplementation. For most gastrointestinal symptoms reported, both severity and frequency of symptoms decreased with time. Finally, dietary supplementation did not affect rating of the symptoms less commonly reported with alli® treatment (data not shown).

### Fecal parameters

**Fig. 3:**
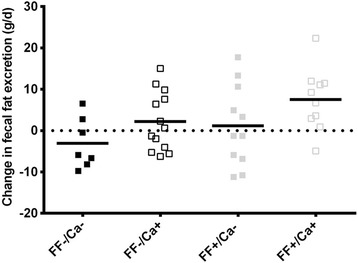
Fecal fat excretion. Individual and mean change in fecal fat excretion (g/d) from baseline (orlistat treatment only) to week 4 (orlistat plus placebo (FF-/Ca-) or flaxseed fibers (FF) and/or dairy Ca (Ca) supplements). * Indicate a significant effect of Ca (*p* = 0.02) and # indicate a significant effect of FF (*p* = 0.04) compared to control in an ANCOVA

In total, complete fecal collections during week 4 were received from 39 participants. None of the fecal parameters differed between treatment groups at baseline (*P* > 0.30) (Table 2). Supplementation with FF, but not Ca, tended to increase the number of defecations (*P* = 0.07). This effect was attenuated after adjustment for compliance (*P* = 0.096), which may be explained by a positive correlation between defecation frequency and compliance (*R* = 0.47; *P* = 0.003). Fecal wet weight increased with supplementation of Ca (*P* = 0.04), and tended also to increase with FF (*P* = 0.09); however % DM was unaffected by both Ca and FF. Both FF and Ca supplementation increased fecal fat excretion (*P* = 0.02 and *P* = 0.04, respectively). Although fecal fat excretion increased by ~100% in the FF+/Ca + group, but only by ~12% in the FF-/Ca + group (Table 2, Fig. 3), no interaction between FF and Ca was observed (*P* = 0.65). When expressed relative to food intake, the proportion of fat excreted tended to increase with both FF and Ca (*P* = 0.08 and *P* = 0.07, respectively).

**Table 2:** Fecal parameters before and after 4 weeks supplementation with flaxseed fibers (FF) and/or dairy calcium (Ca) in addition to alli®^*^

						*P*-values^**^	
	FF-		FF+			Intervention	
	Ca- (*n* = 6)	Ca + (*n* = 13)	Ca- (*n* = 10)	Ca + (*n* = 10)	Baseline	FF	Ca
Defecation frequency (n/d)							
Baseline	1.43 ± 0.17	1.78 ± 0.32	1.21 ± 0.15	1.21 ± 0.11	0.50	0.07	0.79
Week 4	1.24 ± 0.14	1.47 ± 0.26	1.24 ± 0.10	1.39 ± 0.18			
Fecal wet weight (g/d)							
Baseline	169.8 ± 45.9	184.9 ± 31.2	141.5 ± 35.6	152.4 ± 35.6	0.81	0.09	0.04*
Week 4	129.5 ± 36.5	187.1 ± 24.8	144.7 ± 28.3	199.6 ± 28.3			
Fecal dry matter (%)							
Baseline	27.6 ± 2.2	28.7 ± 1.4	30.9 ± 1.5	30.8 ± 1.8	0.56	0.78	0.54
Week 4	29.8 ± 1.8	29.0 ± 1.2	30.5 ± 1.1	30.3 ± 1.7			
Fecal fat excretion (g/d)							
Baseline	17.7 ± 3.2	18.6 ± 2.2	18.6 ± 2.5	15.3 ± 2.5	0.75	0.02*	0.04*
Week 4	15.4 ± 4.3	20.8 ± 2.9	21.0 ± 3.3	25.6 ± 3.3			
Fecal fat excretion (%)							
Baseline	24.5 ± 6.3	31.7 ± 4.3	32.1 ± 4.9	24.9 ± 3.8	0.38	0.08	0.07
Week 4	24.1 ± 10.3	37.1 ± 7.0	36.0 ± 8.0	48.1 ± 8.0			

^*^ *n* = 39; completers only population

^**^P-values refer to an ANCOVA model with adjustment for baseline value, sex and age

### Anthropometric and cardiometabolic risk markers

There was no difference between groups in baseline values, except for HbA1c (*P* = 0.03) (Table 3). Participants in all four groups experienced a weight loss (*P* < 0.01) which was also accompanied by decreased BMI and waist circumference in all groups (*P* < 0.01). An interaction between FF and Ca with respect to waist circumference was seen (*P* = 0.03), as waist circumference tended to decrease more with the combined supplementation of FF and Ca (−5.8 cm) compared to either FF (−3.8 cm) or Ca (−2.7com) alone, but did not differ from control (FF-/Ca-) (−6.1 cm). Systolic blood pressure was not affected by either dietary supplement, whereas diastolic blood pressure was slightly increased with FF supplementation (*P* = 0.03). Blood lipids were unaffected by dietary supplementation, as were markers of glucose homeostasis (glucose, insulin, HOMA-IR and HbA1c). Finally, FF, but not Ca, resulted in increased hsCRP concentrations (*P* = 0.03). When a cut-off value of 10 pg/mL was applied to exclude participants with potential acute infections, the effect of FF was however attenuated (*n* = 31, *P* = 0.21) (data not shown).

**Table 3:** Cardiometabolic risk markers before and after 12 weeks supplementation with flaxseed fibers (FF) and/or dairy calcium (Ca) in addition to alli®^*^

						*P*-values^**^		
		FF-		FF+			Intervention	
		Ca- (*n* = 6)	Ca + (*n* = 12)	Ca- (*n* = 10)	Ca + (*n* = 10)	Baseline	FF	Ca
Body weight (kg)	Baseline	98.9 ± 8.4	103.4 ± 5.2	91.2 ± 3.9	95.6 ± 2.8	0.30		
	Week 12	94.0 ± 9.9	98.9 ± 4.9	87.4 ± 3.8	91.4 ± 2.4		0.46	0.80
WC (cm)	Baseline	111.0 ± 4.8	111.3 ± 3.4	104.5 ± 2.6	104.2 ± 3.2	0.56		
	Week 12	104.9 ± 6.3^ab^	107.5 ± 3.2^a^	101.8 ± 2.7^a^	98.4 ± 2.9^b^		FF × Ca: 0.03*	
Systolic BP (mmHg)	Baseline	116 ± 6	118 ± 2	113 ± 2	123 ± 4	0.52		
	Week 12	116 ± 5	114 ± 3	115 ± 3	129 ± 5		0.58	0.70
Diastolic BP (mmHg)	Baseline	82 ± 3	81 ± 3	78 ± 2	84 ± 2	0.23		
	Week 12	80 ± 4	79 ± 2	75 ± 3	84 ± 2		0.03*	0.61
TAG (mmol/L)	Baseline	0.85 ± 0.33	1.46 ± 0.23	1.65 ± 0.25	0.99 ± 0.25	0.15		
	Week 12	0.85 ± 0.18	1.27 ± 0.14	1.17 ± 0.14	0.92 ± 0.14		0.42	0.56
T-C (mmol/L)	Baseline	4.87 ± 0.39	5.29 ± 0.28	5.31 ± 0.30	5.62 ± 0.30	0.52		
	Week 12	2.39 ± 0.38	4.94 ± 0.27	4.95 ± 0.30	5.11 ± 0.30		0.95	0.87
LDL-C (mmol/L)	Baseline	2.94 ± 0.33	3.24 ± 0.24	3.31 ± 0.26	3.28 ± 0.26	0.82		
	Week 12	2.52 ± 0.30	3.09 ± 0.21	3.12 ± 0.24	2.98 ± 0.24		0.98	0.62
HDL-C (mmol/L)	Baseline	1.45 ± 0.10	1.30 ± 0.07	1.18 ± 0.06	1.65 ± 0.09	0.17		
	Week 12	1.35 ± 0.15	1.15 ± 0.05	1.18 ± 0.06	1.52 ± 0.08		0.19	0.81
Glucose (mmol/L)	Baseline	5.38 ± 0.19	5.34 ± 0.13	5.24 ± 0.15	5.42 ± 0.15	0.84		
	Week 12	5.29 ± 0.20	5.34 ± 0.14	5.30 ± 0.16	5.19 ± 0.16		0.40	0.32
Insulin (pmol/L)	Baseline	83.9 ± 26.6	103.3 ± 18.8	102.4 ± 20.6	73.4 ± 20.6	0.67		
	Week 12	62.0 ± 38.3	114.6 ± 24.7	83.0 ± 27.1	70.0 ± 27.1		0.52	0.25
HOMA-IR	Baseline	2.92 ± 0.58	3.54 ± 0.63	3.65 ± 1.21	2.59 ± 0.41	0.68		
	Week 12	2.10 ± 0.46	3.99 ± 1.31	2.96 ± 0.80	2.34 ± 0.47		0.97	0.67
HbA1c (%)	Baseline	5.32 ± 0.05^a^	5.59 ± 0.08^b^	5.50 ± 0.09^ab^	5.57 ± 0.12^b^	0.03*		
	Week 12	5.38 ± 0.10	5.48 ± 0.08	5.48 ± 0.09	5.47 ± 0.10			
hsCRP (pg/mL)	Baseline	3.23 ± 1.76	3.11 ± 1.15	4.81 ± 1.43	7.61 ± 2.41	0.92		
	Week 12	3.93 ± 0.70	2.07 ± 0.45	5.06 ± 1.61	5.80 ± 1.90		0.03*	0.61

Abbreviations: *BMI* body mass index, *BP* blood pressure, *HDL-C* HDL cholesterol, *hsCRP* high sensitivity C-reactive protein, *HOMA-IR* Homeostasis Model of Assessment - Insulin Resistance, *HbA1c* glycated hemoglobin, *LDL-C* LDL cholesterol, *TAG* triacylglycerol, *T-C* total cholesterol, *WC* waist circumference

^*^ *n* = 39; completers only population

^**^ *P*-values refer to an ANCOVA model with adjustment for baseline value, sex and age. ﻿Columns with different superscript letters are significantly different; *P<0.05*

## Discussion

We tested the hypothesis that supplementation with FF and/or Ca would alleviate orlistat-induced gastrointestinal symptoms as a consequence of intestinal sequestering of free oil, possibly also accompanied by increased fecal fat excretion. We were not able to demonstrate an improvement in gastrointestinal symptom profile; but interestingly, we found that combined supplementation of FF and Ca resulted in a marked increase in fecal fat excretion compared to control (FF-/Ca-), which was not observed with either FF or Ca alone. Thus, a combined supplementation of FF and Ca may aid weight loss as a consequence of fecal fat losses beyond the effect of orlistat.

The increased fecal fat excretion with both FF and Ca, and in particular the combination of the two, was not accompanied by changes in ratings of gastrointestinal symptoms apart from a tendency towards an increase in severity, but not frequency, of diarrhea with Ca. The fecal fat excretion was ~10 g/d higher with FF and Ca (~25 g/d) compared to fecal fat excretion with orlistat alone (~15 g/d). which is a marked increase from the ~155 g/d of fecal fat excretion with orlistat alone. Orlistat diminishes fat absorption by inhibiting the activity of the gastrointestinal lipase resulting in an increased amount of intact non-hydrolyzed triacylglycerols in the intestine. Ca is proposed to form insoluble soaps with these triacylglycerols [25], which may occur more distally, thus, orlistat may not affect the ability of Ca to form soaps, but rather result in an increased amount of lipids available for soap formation. FF are highly viscous dietary fibers [26] and as such, they are proposed to increase the viscosity of the intestinal contents which both thickens the unstirred water-layer along the mucosal barrier as well as slows digestive enzyme activity, including lipases [14]. Thus, both mechanisms of action appear to act in combination with orlistat, which does not appear to affect magnitude of effect of the supplements. Orlistat alone resulted in a fecal fat excretion of 25-30% of intake, and fecal fat excretion depends on dose of orlistat, although not in a linear manner [4]. Thus, components exerting their effects via different mechanisms may exceed this plateauing at a given dose, as suggested by our results. However, the combination of FF and Ca without orlistat treatment has not been tested; thus it is unknown whether orlistat may in fact facilitate some of the effects observed by increasing the intestinal triacylglycerol content.

As previously reported, attrition rates are high for orlistat treatment [3], and the present study was no exception. Overall, 33% of the participants dropped out of the study, and ~40% (6 participants of which 5 dropped out after randomization) of these were directly related to gastrointestinal side effects. Only one participant receiving Ca dropped out, whereas three participants receiving FF dropped out indicating that the supplements affected gastrointestinal symptoms differently; however the study was too small to assess differences in attrition rates and thus this warrants further investigations. Nonetheless, the compliance to alli® was very high, which may be related to the fact that a few participants consumed a larger amount of alli® than instructed, likely because they know that a higher dose of orlistat could be taken without increased risk. The compliance to the dietary supplements was lower, likely due to its effect on palatability of the foods in which they were applied. However, a large proportion of the drop outs occurred during week 4, where dietary records and fecal collections took place. Thus, some of the dropouts may be related to the cumbersome procedures the participants were requested to follow, rather than the intervention itself.

Surprisingly, FF supplementation resulted in increased diastolic blood pressure and hsCRP, which is in contrast to previous reports on anti-inflammatory effects of flaxseeds among obese individual [26, 27]. Mean baseline blood pressure was within the normal range in the current study, whereas mean hsCRP concentrations were very high indicating an at risk population. However, in the subset of participants with hsCRP < 10 pg/mL, the effect was attenuated; thus the pro-inflammatory effect of FF is not a robust finding. Moreover, we conducted multiple comparisons i.e. the secondary end-points without adjustments, which makes it likely that these findings are spurious and a matter of chance. The findings need to be replicated in independent studies.

Overall, compliance with both orlistat and dietary supplements was good, but tended to be best with Ca, whereas larger variations were observed in the FF supplemented groups. FF are viscous dietary fibers, and form highly viscous solutions immediately upon hydration. Thus the distribution of the FF as powders to be dissolved in liquids or sprayed on foods, e.g. yoghurt, may have given rise to less palatable foods, which may have affected compliance. This is not the case for Ca, which is solubilized and affects the taste of the food, but not the texture and thus it may have been better tolerated.

Few studies have examined the effect of dietary components on orlistat-induced fecal fat excretion and gastrointestinal symptoms. In contrast to the current findings, 6 g of psyllium given three times daily in conjunction with 120 mg orlistat was found to decrease occurrence of gastrointestinal events compared to placebo in a crossover study in 30 obese participants; however, fecal fat excretion was not assessed [17]. Psyllium and FF are both highly viscous dietary fibers, but the dose of psyllium was ~3 times higher than the FF dose used in the present study, which may explain why the results differ. Furthermore, a crossover design may be preferable when assessing subjective ratings of symptoms as inter-individual variation then can be eliminated. In the present study, we were not able to control type of fat; however, amount of fat consumed did not differ between groups and only decreased by few grams during the study, thus we do not believe fat intake to have affected our results, where patterns of relative fecal fat excretion (% of intake) followed that of fecal fat excretion in absolute amounts. A highly controlled study revealed that foods high in dietary fibers given in conjunction with 3 × 80 mg orlistat did not affect fecal fat excretion patterns differently than low-fiber foods, whereas extracellular fat (rendered lard) increased fecal fat excretion compared to intracellular fat (un-rendered lard) [16]. These results suggest that fat type in the background diet may be of great importance for the efficacy of orlistat whereas the content of dietary fibers, in natural food matrices, may vary considerably without affecting fat absorption.

Major strengths of the present study are the 2x2 factorial design, which enabled us to study interactions; that the intervention was well-controlled and resulted in a high compliance in all participants; and that that both gastrointestinal symptoms and fecal fat excretion were assessed and could thus be compared. However, the small sample size, partly caused by a large attrition rate, limits our ability to make firm conclusions based on these results.

## Conclusions

The present findings do not support alleviation of orlistat-induced gastrointestinal side effects by supplementation of FF and/or Ca as hypothesized. However, fecal fat excretion was increased with both FF and Ca in the absence of a worsening of symptoms and to the greatest extent when both FF and Ca were consumed indicating a substantial additive effect.

## Additional file

Additional file 1: Tables S1, S2 and S3. (DOCX 36 kb)

## Abbreviations

Ca: Dairy calcium in the form of Capolac®
Ca-: Supplementation without 1200 mg dairy calcium
Ca+: Supplementation with 1200 mg dairy calcium
ER: Estimated energy requirement
FF: Flaxseed fiber
FF-: Supplementation without 5 g flaxseed fiber
FF+: Supplementation with 5 g flaxseed fiber
FFQ: Food frequency questionnaire
HbA1c: Hemoglobin A1C
HOMA-IR: HOMA index of insulin resistance
hsCRP: Serum high sensitivity CRP
