# Supplementary material for: Supplementation with dairy calcium and/or flaxseed fibers in conjunction with orlistat augments fecal fat excretion without altering ratings of gastrointestinal comfort
Source: Nutr Metab (Lond). 2017 Feb 7;14:13. doi: 10.1186/s12986-017-0164-8 (PMC5297204; doi:10.1186/s12986-017-0164-8)
Supplement: Additional file 1: — Tables S1, S2 and S3. (DOCX 36 kb) [file 12986_2017_164_MOESM1_ESM.docx]

**Additional files**

**Table S1** Dietary intake before and after 4 weeks supplementation with flaxseed fibers (FF) and/or dairy calcium (Ca) in addition to alli®.^1^

|  |  | |  | | P-values ^2^ | | |
| --- | --- | --- | --- | --- | --- | --- | --- |
|  | **FF-** | | **FF+** | |  | **Intervention** | |
|  | **Ca- (n=6)** | **Ca+ (n=13)** | **Ca- (n=10)** | **Ca+ (n=10)** | **Baseline** | **FF** | **Ca** |
| Energy intake (kJ/d)  Run in  Week 4 | 7139 ± 746  6736 ± 656 | 6884 ± 308  6374 ± 463 | 6532 ± 337  6037 ± 260 | 6529 ± 428  6834 ± 529 | 0.51 | 0.39 | 0.73 |
| Fat intake (g/d)  Run in  Week 4 | 74.4 ± 7.8  72.5 ± 9.4 | 63.3 ± 3.8  59.3 ± 4.3 | 58.2 ± 4.0  59.4 ± 5.0 | 65.5 ± 4.1  63.3 ± 6.0 | 0.21 | 0.63 | 0.76 |
| Fat intake (E%)  Run in  Week 4 | 39.2 ± 3.2  39.6 ± 2.8 | 33.9 ± 1.1  34.9 ± 1.2 | 32.9 ± 1.6  36.0 ± 2.0 | 37.3 ± 1.2  34.0 ± 1.2 | 0.80 | 0.69 | 0.93 |
| Protein intake (E%)  Run in  Week 4 | 22.6 ± 1.1  19.6 ± 1.3 | 21.4 ± 0.7  21.4 ± 1.0 | 21.1 ± 1.2  21.8 ± 1.1 | 21.1 ± 0.9  21.3 ± 0.8 | 0.50 | 0.31 | 0.48 |
| Carbohydrate intake (E%)  Run in  Week 4 | 36.9 ± 4.5  40.4 ± 2.6 | 43.5 ± 1.8  41.8 ± 1.4 | 44.0 ± 1.9  41.4 ± 2.1 | 40.8 ± 1.4  43.4 ± 1.3 | 0.72 | 0.35 | 0.53 |

^1^ n=39, completers only population.

^2^ P-values refer to an ANCOVA model with adjustment for run-in value, sex and age.

**Table S2** Severity of gastrointestinal symptoms before and averaged over 12 weeks supplementation with flaxseed fibers (FF) and/or dairy calcium (Ca) in addition to alli®.^1^ The lower the score the less frequent or severe the symptom.

|  | |  | |  | | P-values^2^ | | | | |  |
| --- | --- | --- | --- | --- | --- | --- | --- | --- | --- | --- | --- |
|  | | **FF-** | | **FF+** | |  | | **Intervention** | | | |
|  | | **Ca- (n=6)** | **Ca+ (n=12)** | **Ca- (n=10)** | **Ca+ (n=10)** | **Baseline** | | **FF** | | **Ca** | |
| Diarrhea (mm) | Baseline  Week 2-12 | 6.3 ± 7.5  11.1 ± 7.8^ab^ | 9.4 ± 5.1  11.7 ± 5.5^b^ | 9.6 ± 5.6  4.8 ± 6.1^ab^ | 3.7 ± 5.6  22.9 ± 6.1^ac^ | 0.86 | FF × Ca: 0.08 | | | | |
| Flatus (mm) | Baseline  Week 2-12 | 47.5 ± 10.9  32.9 ± 10.9 | 39.9 ± 7.7  34.4 ± 7.7 | 33.2 ± 8.5  26.8 ± 8.4 | 39.9± 8.5  36.0 ± 8.4 | 0.78 | 0.69 | | 0.35 | | |
| Flatus w/ discharge (mm) | Baseline  Week 2-12 | 1.5 ± 7.4  13.4 ± 6.7 | 13.9 ± 5.2  19.3 ± 4.7 | 1.3 ± 5.8  7.9 ± 5.2 | 20.4 ± 5.8  10.5 ± 5.2 | 0.08 | 0.91 | | 0.28 | | |
| Fecal urgency (mm) | Baseline  Week 2-12 | 14.2 ± 9.9  13.9 ± 7.2 | 11.9 ± 7.0  20.2 ± 5.1 | 10.7 ± 7.7  10.5 ± 5.6 | 20.2 ± 7.7  23.8 ± 5.6 | 0.82 | 0.91 | | 0.14 | | |
| Fecal incontinence (mm) | Baseline  Week 2-12 | 2.3 ± 1.0  4.7 ± 4.9 | 1.3 ± 0.7  6.2 ± 3.5 | 0.9 ± 0.8  2.9 ± 3.9 | 1.3 ± 0.8  13.4 ± 3.9 | 0.76 | 0.42 | | 0.17 | | |
| Loose stools (mm) | Baseline  Week 2-12 | 45.2 ± 12.0  28.9 ± 8.9 | 40.5 ± 8.5  34.0 ± 6.3 | 29.4 ± 9.3  17.5 ± 6.9 | 44.2 ± 9.3  32.3 ± 6.9 | 0.65 | 0.53 | | 0.33 | | |
| Oily stools (mm) | Baseline  Week 2-12 | 40.5 ± 11.8  21.6 ± 9.6 | 27.6 ± 8.3  27.1 ± 6.8 | 14.9 ± 9.1  14.5 ± 7.4 | 32.7 ± 9.1  26.8 ± 7.4 | 0.34 | 0.96 | | 0.38 | | |
| Frequent stools (mm) | Baseline  Week 2-12 | 21.1 ±24.4 | 16. ± 20.2 | 21.7 ± 21.2 | 16.8 ± 16.5 | 0.76 | 0.25 | | 0.24 | | |
|  |  | 18.4 ± 3.5 | 19.5 ± 2.8 | 17.9 ± 2.6 | 24.8 ± 6.2 |  |  |  |  |  |  |
| Abdominal pain (mm) | Baseline  Week 12 | 15.3 ± 20.5^a^ | 2.6 ± 5.2^b^ | 6.3 ± 14.9^ab^ | 7.3 ± 16.9^ab^ | **<0.01** | 0.66 | | 0.61 | | |
|  |  | 9.2 ± 3.2 | 9.0 ± 2.6 | 6.0 ± 1.3 | 8.9 ± 3.1 |  |  |  |  |  |  |
| Composite score (mm) | Baseline  Week 2-12 | 22.0 ± 5.1  17.9 ± 6.1 | 20.4 ± 3.6  20.8 ± 4.3 | 15.3 ± 4.2  12.1 ± 4.7 | 21.2 ± 4.0  22.8 ± 4.7 | 0.68 | 0.99 | | 0.40 | | |

^1^ n=39; completers only population.

^2^ P-values refer to an ANCOVA model with adjustment for baseline value, sex and age.

**Table S3** Frequency of gastrointestinal symptoms before and averaged over 12 weeks supplementation with flaxseed fibers (FF) and/or dairy calcium (Ca) in addition to alli®.^1^ The lower the score the less frequent or severe the symptom.

|  | |  | | | |  | | | P-values ^2^ | | | |  | |
| --- | --- | --- | --- | --- | --- | --- | --- | --- | --- | --- | --- | --- | --- | --- |
|  | | **FF-** | | | | **FF+** | | |  | | **Intervention** | | | |
|  | | **Ca- (n=6)** | | **Ca+ (n=12)** | | **Ca- (n=10)** | **Ca+ (n=10)** | | **Baseline** | | **FF** | | | **Ca** |
| Diarrhea (mm) | Baseline  Week 2-12 | 6.2 ± 8.5  9.5 ± 7.5 | 13.0 ± 6.0  12.6 ± 5.3 | | 9.3 ± 6.6  11.6 ± 5.8 | | 1.7 ± 6.6  21.8 ± 5.8 | 0.65 | | 0.73 | | 0.13 | | |
| Flatus (mm) | Baseline  Week 2-12 | 36.0 ± 13.9  31.6 ± 11.3 | 53.8 ± 9.8  38.0 ± 8.0 | | 32.4 ± 10.8  25.6 ± 8.7 | | 46.3 ± 10.8  33.9 ± 8.7 | 0.48 | | 0.80 | | 0.98 | | |
| Flatus w/ discharge (mm) | Baseline  Week 2-12 | 1.8 ± 9.7  21.3 ± 8.1 | 14.1 ± 6.9  21.5 ± 5.7 | | 1.4 ± 7.5  8.1 ± 6.5 | | 29.0 ± 7.5  11.5 ± 6.3 | 0.06 | | 0.85 | | 0.14 | | |
| Fecal urgency (mm) | Baseline  Week 2-12 | 7.2 ± 10.6  16.2 ± 7.8 | 11.7 ± 8.2  12.5 ± 6.1 | | 10.6 ± 7.5  23.2 ± 5.5 | | 24.1 ± 8.1  24.0 ± 6.0 | 0.53 | | 0.59 | | 0.25 | | |
| Fecal incontinence (mm) | Baseline  Week 2-12 | 2.8 ± 1.1  5.3 ± 5.1 | 1.8 ± 0.8  7.5 ± 3.6 | | 0.8 ± 0.9  3.2 ± 4.0 | | 1.0 ± 0.8  12.8 ± 4.0 | 0.44 | | 0.33 | | 0.13 | | |
| Loose stools (mm) | Baseline  Week 2-12 | 37.3 ± 13.9  24.3 ± 9.5 | 37.3 ± 9.8  35.7 ± 6.7 | | 25.6 ± 10.8  15.4 ± 7.4 | | 45.2 ± 10.8  33.6 ± 7.4 | 0.65 | | 0.47 | | 0.12 | | |
| Oily stools (mm) | Baseline  Week 2-12 | 34.5 ± 15.0  23.5 ±10.8 | 41.4 ± 10.6  32.4 ± 7.6 | | 19.1 ± 11.6  16.8 ± 8.3 | | 39.1 ± 11.6  27.5 ± 8.3 | 0.52 | | 0.69 | | 0.81 | | |
| Frequent stools (mm) | Baseline  Week 2-12 | 13.3 ± 13.3 | 14.3 ± 19.3 | | 16.7 ± 30.0 | | 10.2 ± 14.7 | 0.16 | | 0.63 | | 0.22 | | |
|  |  | 17.2 ± 4.0 | 20.4 ± 4.9 | | 14.7 ± 2.7 | | 11.5 ± 5.9 |  |  |  |  |  |  |  |
| Abdominal pain (mm) | Baseline  Week 12 | 14.7 ± 20.5^a^ | 3.0 ± 5.8^b^ | | 7.4 ± 18.0^ab^ | | 7.1 ± 16.2^ab^ | **<0.01** | | 0.94 | | 0.50 | | |
|  |  | 9.5 ± 3.4 | 11.8 ± 3.0 | | 8.0 ± 2.1 | | 8.8 ± 3.0 |  |  |  |  |  |  |  |
| Composite score (mm) | Baseline  Week 2-12 | 17.1 ± 6.0  18.3 ± 6.2 | 23.1 ± 4.0  23.4 ± 4.4 | | 13.9 ± 4.3  12.1 ± 4.9 | | 23.0 ± 4.3  22.2 ± 4.8 | 0.39 | | 0.50 | | 0.78 | | |

^1^ n=39; completers only population.

^2^ P-values refer to an ANCOVA model with adjustment for baseline value, sex and age.
